# Chronic exposure to diesel exhaust may cause small airway wall thickening without lumen narrowing: a quantitative computerized tomography study in Chinese diesel engine testers

**DOI:** 10.1186/s12989-021-00406-1

**Published:** 2021-03-25

**Authors:** Hong Liu, Jianyu Li, Qianli Ma, Jinglong Tang, Menghui Jiang, Xue Cao, Li Lin, Nan Kong, Shanfa Yu, Akshay Sood, Yuxin Zheng, Shuguang Leng, Wei Han

**Affiliations:** 1 Department of Respiratory and Critical Care Medicine, Qingdao Municipal Hospital, School of Medicine, Qingdao University, Qingdao, 266021 China; 2 Department of Occupational and Environmental Health, School of Public Health, Qingdao University, Qingdao, 266021 Shandong China; 3 Henan Institute of Occupational Medicine, Zhengzhou, Henan China; 4 Department of Internal Medicine, School of Medicine, University of New Mexico, Albuquerque, NM 87131 USA; 5 Cancer Control and Population Sciences, University of New Mexico Comprehensive Cancer Center, Albuquerque, NM 87131 USA

**Keywords:** Diesel exhaust, Airway wall thickening, Carbon content in airway macrophage, Spirometry, Mediation effect

## Abstract

**Background:**

Diesel exhaust (DE) is a major source of ultrafine particulate matters (PM) in ambient air and contaminates many occupational settings. Airway remodeling assessed using computerized tomography (CT) correlates well with spirometry in patients with obstructive lung diseases. Structural changes of small airways caused by chronic DE exposure is unknown. Wall and lumen areas of 6th and 9th generations of four candidate airways were quantified using end-inhalation CT scans in 78 diesel engine testers (DET) and 76 non-DETs. Carbon content in airway macrophage (CCAM) in sputum was quantified to assess the dose-response relationship.

**Results:**

Environmental monitoring and CCAM showed a much higher PM exposure in DETs, which was associated with higher wall area and wall area percent for 6th generation of airways. However, no reduction in lumen area was identified. No study subjects met spirometry diagnosis of airway obstruction. This suggested that small airway wall thickening without lumen narrowing may be an early feature of airway remodeling in DETs. The effect of DE exposure status on wall area percent did not differ by lobes or smoking status. Although the trend test was of borderline significance between categorized CCAM and wall area percent, subjects in the highest CCAM category has a 14% increase in wall area percent for the 6th generation of airways compared to subjects in the lowest category. The impact of DE exposure on FEV1 can be partially explained by the wall area percent with mediation effect size equal to 20%, P_perm_ = 0.028).

**Conclusions:**

Small airway wall thickening without lumen narrowing may be an early image feature detected by CT and underlie the pathology of lung injury in DETs. The pattern of changes in small airway dimensions, i.e., thicker airway wall without lumen narrowing caused by occupational DE exposure was different to that (i.e., thicker airway wall with lumen narrowing) seen in our previous study of workers exposed to nano-scale carbon black aerosol, suggesting constituents other than carbon cores may contribute to such differences. Our study provides some imaging indications of the understanding of the pulmonary toxicity of combustion derived airborne particulate matters in humans.

**Supplementary Information:**

The online version contains supplementary material available at 10.1186/s12989-021-00406-1.

## Introduction

International Agency for Research on Cancer escalated the carcinogenicity classification of diesel exhaust (DE) to human carcinogen in 2012 based on sufficient human evidence for lung cancer [1]. In addition to lung cancer, chronic DE exposure has also been linked to many non-malignant pulmonary and extra-pulmonary health effects. DE from on-road vehicles was regarded as the most important source of traffic related fine particulate matter (PM2.5) in urban atmosphere in developed countries [1–3]. DE exposure was also common for workers from multiple occupational settings including underground mining, bridge and tunnel construction, trucking, railroad, etc. [2] DE consists of volatile gases and particulate matters with the latter composed of elemental carbon (EC) cores carrying organic and inorganic pollutants. DE particulates were of nano-size (< 100 nm) and could deposit deep and have long retention time inside the lung [2, 4–6].

Controlled exposure human studies in chamber or panel studies with real-life exposures such as from diesel-powered trains, underground gold mining, and tunnel construction have identified evidence of lung function alterations (e.g., elevated airway resistance and reduced spirometry measurements) due to short-term (i.e., several hours to several days) exposure to DE [7–10]. In addition, cumulative dose of EC exposure during 5-yr follow-up period was associated with a more rapid lung function decline in underground potash salt miners [11]. However, controlled exposure studies are more suitable for detecting acute or subacute responses of exposure, while panel studies using occupational populations suffer from exposure to a complex mixture of air pollutants such as various mine dust, gasoline emission, etc. that may confound the associations between DE exposure and health outcomes. Recently, we characterized a unique occupational cohort of diesel engine testers (DET) who exam the performance of the newly assembled diesel-fueled vehicle engines for quality certification purpose in China [12]. Environmental monitoring at the workplace suggested that DETs were exposed to high levels of PM2.5 (i.e., > 282.3 μg/m^3^) and EC (i.e., > 135.2 μg/m^3^) [12–14]. In addition, DETs had significant reduced spirometry measurements indicative of airway obstruction and serological evidence indicative of Clara cell injury and elevated permeability of acinar airways [15]. However, lung tissue remodeling underlying the observed phenotypic changes in DETs is largely unknown.

Structural changes within airway wall is an important component of lung remodeling commonly seen in patients with asthma and chronic obstructive pulmonary disease (COPD) and pathologically consists of increased basement membrane thickness of airway epithelium, hypertrophy of the smooth muscle cell, and peri-bronchial fibrosis [16]. Airway remodeling parameters obtained through tissue biopsies are related to important pheno- and endo-types in animal and human studies [17–19]. However, human studies had limited availability of lung biopsies for assessing lung remodeling. With the recent technical advancement of quantitative computed tomography (CT) and analytical informatics, morphological changes of intermediate to small airways (e.g., airway wall and lumen thickness) and emphysema-like lung parenchymal impairment could be determined using end-inhalation CT scans in patients with asthma or COPD and correlate well with biopsy findings [16, 20]. With these advanced techniques, we have successfully generate a detailed assessment of airway remodeling in the lungs of carbon black packers based on which small airway wall thickening with lumen narrowing was identified as an early pathological change in the lungs due to inhalation exposure of nano-scale carbon black aerosol [21].

EC constitutes a larger portion of the diesel particulate mass and has been used as an external exposure dosimetry for assessing dose-response of health effects with DE exposure [1, 22]. However, external assessment of inhalation exposure suffers from lack of consideration of inter-individual variations in respiratory physiology and lung defense and clearance capacity. Carbon content in airway macrophages (CCAM) has been used as a quantitative and bio-effective measure for lung EC burden to evaluate individual’s exposure to DE particulates and other carbon containing particulates and has been successfully applied to assess the dose-response with varied health effects [13, 23–31]. Our methodology improvement, i.e., using Saccomano’s fixative to preserve and wash sputum samples made this assay suitable for large-scale epidemiological application and minimized “presumed” interference by pigmented component from cigarette smoke for the identification of black particles in airway macrophages [21, 26].

In this study, we hypothesized that chronic exposure to DE aerosol in DETs may cause airway remodeling as a pathophysiological basis underlying lung function impairment. This was assessed in 78 DETs and 76 non-DETs with chest CT scan data from Henan, China. Airway dimensions were quantified for the 6th and 9th generations of tracheobronchial tree, the very beginning segments of small airways. The findings of this study provided the first comprehensive quantification of airway remodeling in workers exposed to DE aerosol. A comparison of the patterns of airway dimension changes between DE and nano-scale carbon black exposures [21] may shed light on the understanding of the pulmonary toxicity of different constituents of combustion derived airborne particulate matters in humans.

## Results

### Exposure assessment

**Fig. 1:**
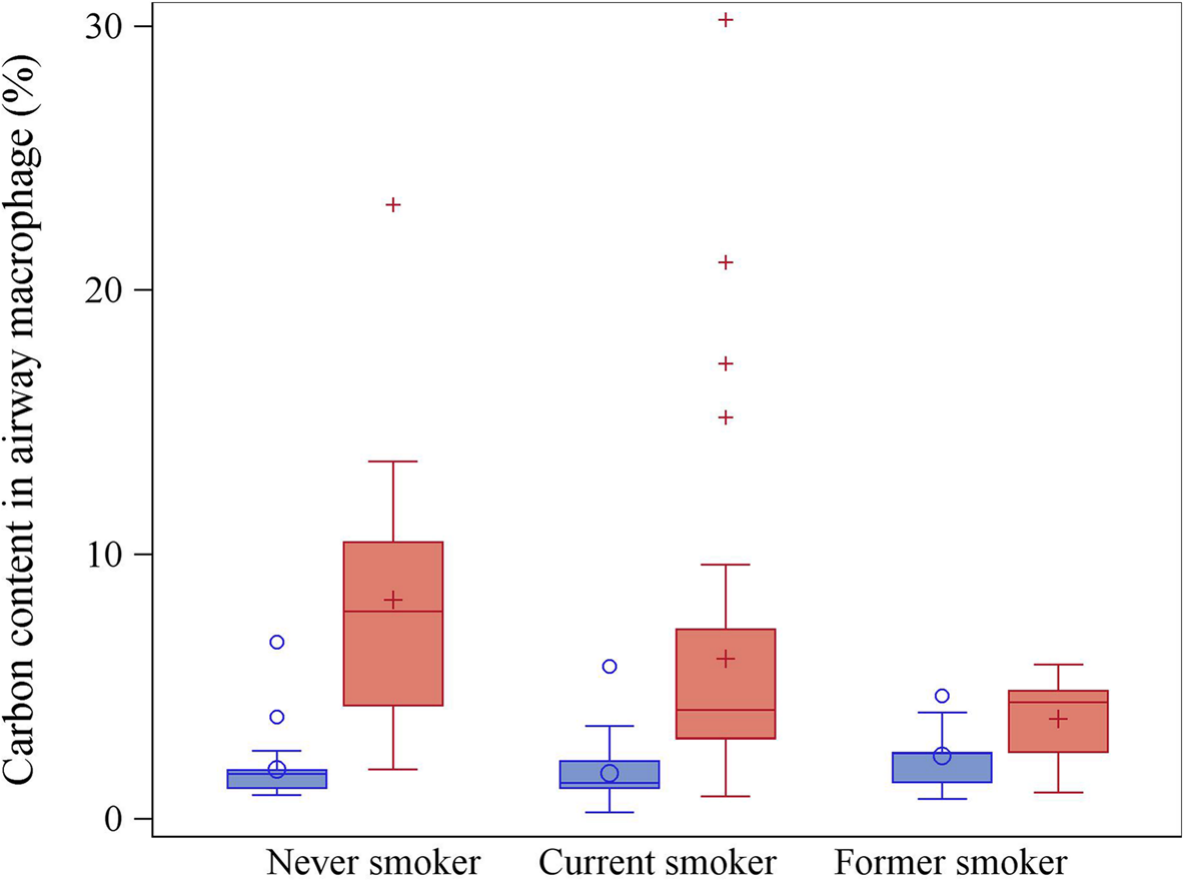
The distribution of CCAM in non-DETs (*n* = 53) and DETs (n = 53) by smoking status. CCAM in DETs was significantly higher than that seen in non-DETs regardless of smoking status (all Ps < 0.04). Moreover, CCAM did not vary by smoking status in either non-DETs (*P* = 0.37) or DETs (*P* = 0.11) using Wilcoxon rank sum test. Red box represents DET, Blue box represents non-DET. The five horizontal bars from bottom to top represent the minimum, first quartile, median, third quartile, and maximum. Symbols (e.g., ○ and +) represent means inside the box and outliers outside the box. CCAM = carbon content in airway macrophage; DET = diesel engine tester

Up to 84.3% DE particulates had aerodynamic diameter less than 100 nm with 100% particulates less than 1000 nm, thus DE particles fall in the ultrafine particle range [14]. Geometric mean of PM2.5 level in DET work shop was 430.8 μg/m^3^, which was 3.3-fold higher than levels seen in the reference areas (130.0 μg/m^3^, Table 1). PM2.5 exposure of the reference areas was PM2.5 levels calculated from five air samples collected from the water utility company areas in October 2018 when the CT study was conducted. These values were used to represent the background particulate matter levels of this study and were similar to what was reported for Luoyang city in the national air quality surveillance system of China during the period of time when this study was conducted. Our previous field surveys identified higher PM2.5 levels in DET workshops (267.5–495.9 μg/m^3^) than reference areas (83.5–91.9 μg/m^3^) as well as a high non-EC component in PM2.5 (52.1–72.1%) collected from DET workshops [12, 14, 32]. CCAM in DETs was significantly higher than that seen in non-DETs regardless of smoking status (all Ps < 0.04. Table 1 and Fig. 1). Moreover, CCAM did not vary by smoking status in non-DETs (*P* = 0.37) or DETs (*P* = 0.11) using Wilcoxon rank sum test (Fig. 1).

**Table 1:** Demographic characteristics, clinical variables, and particulate matter exposure of non-DET (*n* = 76) and DET (*n* = 78)

Variable	Non-DET	DET	*P* values
N	76	78	
Demographics			
Age (yr, M ± SD)	36.4 ± 10.9	36.2 ± 8.0	0.89^a^
Male sex (n, %)	76, 100	78, 100	
Height (cm, M ± SD)	172.3 ± 5.9	173.0 ± 5.3	0.41^a^
BMI (kg/m^2^, M ± SD)	25.7 ± 4.4	25.7 ± 3.4	0.99^a^
Smoking Status			0.004^b^
Never smoker (n, %)	33, 43.4	15, 19.2	
Current smoker (n, %)	34, 44.7	54, 69.2	
Former smoker (n, %)	9, 11.8	9, 11.5	
Packyears in ever smokers (Mdn, Q1 - Q3)	13.5, 6.0–24.0	6.5, 2.6–15.0	0.008^c^
Particulate matter exposure			
DE exposure history (yr, M ± SD)	–	11.7 ± 6.3	
PM_2.5_ (μg/m^3^, n, GSM, GSD)	5, 130.0, 1.2	4, 430.8, 2.3	
CCAM (%, n, Mdn, Q1 - Q3)	53, 1.67, 1.20–2.19	53, 4.29, 3.07–6.49	< 0.001^c^
Self-reported symptom in past one year			
Coughing for ≥3 mo (n, %)	0, 0.0	2, 2.6	0.50^b^
Phlegm for ≥3 mo (n, %)	1, 1.3	6, 7.7	0.12^b^
Radiographic diagnosis			
Emphysema (n, %)	4, 5.3	1, 1.3	0.21^b^
Pulmonary fibrosis (n, %)	0, 0.0	0, 0.0	NC
GGO (n, %)	0, 0.0	0, 0.0	NC
Total lung volume (ml, M ± SD)	5054.1 ± 954.4	5020.4 ± 969.0	0.83^a^
Spirometry			
FEV1 (L/s, M ± SD)	3.64 ± 0.62	3.53 ± 0.42	0.020^d^
FEV1% predicted (%, M ± SD)	98.02 ± 10.75	94.15 ± 11.33	0.025^d^
FVC (L, M ± SD)	4.15 ± 0.69	4.05 ± 0.52	0.039^d^
FVC % predicted (%, M ± SD)	102.18 ± 12.76	99.06 ± 10.89	0.040^d^
FEV1/FVC (%, M ± SD)	87.71 ± 5.41	87.38 ± 5.18	0.78^d^
MMF (L/s, M ± SD)	4.30 ± 1.00	4.15 ± 0.87	0.26^d^
MMF % predicted (%, M ± SD)	91.34 ± 20.08	87.88 ± 21.45	0.32^d^
FEF25 (L/s, M ± SD)	7.22 ± 1.45	6.67 ± 1.19	0.010^d^
FEF25 percent predicted (%, M ± SD)	89.02 ± 17.73	81.79 ± 15.03	0.010^d^
FEF50 (L/s, M ± SD)	4.97 ± 1.29	4.74 ± 1.02	0.165^d^
FEF50 percent predicted (%, M ± SD)	87.07 ± 21.51	82.75 ± 18.99	0.186^d^
FEF75 (L/s, M ± SD)	2.25 ± 0.74	2.17 ± 0.69	0.444^d^
FEF75 percent predicted (%, M ± SD)	78.09 ± 24.34	75.49 ± 26.81	0.580^d^

Definition of abbreviations: *BMI* body mass index, *CCAM* carbon content in airway macrophage, *DET* diesel engine tester, *FEV1* forced expiratory volume in 1 s, *FEF25* Forced expiratory flow rate at 25% vital capacity, *FEF50* Forced expiratory flow rate at 50% vital capacity, *FEF75* Forced expiratory flow rate at 75% vital capacity, *FVC* forced vital capacity, *GGO* ground glass opacity, *GSD* geometric standard deviation,; *GSM* geometrical mean, *L/S* liters per second, *M* mean, *Mdn* median, *MMF* maximal mid-expiratory flow, *NC* not calculated, *PM*_*2.5*_ fine particulate matter, *Q1* lower quartile, *Q3* upper quartile, *SD* standard deviation

^a^ Student t-test

^b^ Chi-square test. Fisher exact test was used for Coughing for ≥3 mo, Phlegm for ≥3 mo, Emphysema, Pulmonary fibrosis, and GGO

^c^ Wilcoxon rank sum test

^d^ Generalized linear model assessed the differences of spirometry between non-DETs and DETs with adjustment of age, height, BMI, and smoking history

### Characteristics of study subjects

In the current study, twenty-four subjects had incomplete data for smoking history and/or end-inhalation lung CT data and were excluded from this study. Spirometry data and CCAM were of no difference (all Ps > 0.30) between the 154 subjects in the study and 24 subjects excluded from current study by DE exposure status, suggesting that our analyses were less likely to be biased by exclusion of these 24 subjects. All study subjects are males. Age and BMI are comparable between the two study groups. DETs were more likely to be current smokers but with less packyears in ever smokers than non-DETs (Table 1). DETs had a median exposure history of 11.7 years. Compared to non-DETs, DETs had lower forced expiratory volume in 1 s (FEV1), forced vital capacity (FVC), and forced expiratory flow rate at 25% vital capacity (FEF25) for both raw and percent predicted values. CT-assessed total lung volume was not different between the two groups, neither for radiographic diagnoses or symptoms. The CT images of four non-DETs and 1 DET showed obvious emphysema characteristics defined as the presence of localized areas of abnormally low attenuation without surrounding walls or with very thin walls [33]. We further calculated the emphysematous volume score (the percentage of volume of low attenuation areas below − 950 Hounsfield units to the volume of total lung capacity on full-inspiration CT images) of the these five people, and the values were 4.56, 4.14, 1.91, 1.64, and 0.08% with the first four values ranking the top in all study subjects. Assessment of indoor winter heating method, type of cooking fuel, kitchen ventilation, and residential distance to major roads defined as two lanes in each direction did not identify any differences between the two study groups (data not shown). In addition, there were seven national regulatory monitor sites for air quality in Luoyang City. We calculated the daily PM2.5 in October 2018 when this study was conducted and assigned the values obtained from the nearest station based on the distance to residential address to each study subject. The average PM2.5 levels in residential area were 53.75 ± 5.48 μg/m^3^ in DETs versus 53.61 ± 5.56 μg/m^3^ in non-DETs (*P* = 0.81). The average PM10 levels in residential area were 102.63 ± 7.74 μg/m^3^ in DETs versus 102.11 ± 8.01 μg/m^3^ in non-DETs (*P* = 0.48). Thus, there is little evidence supporting that our results could be confounded by ambient air pollution in residential area.

### Associations between clinical characteristics and airway dimensions

Airway measurements from 43 quantitative CTs randomly selected were measured independently by a second analyst with good to excellent intra-class correlation coefficients [21]. Airways of 6th and 9th generations of tracheobronchial tree had diameters of 4.5–5.6 mm and 2.6–3.0 mm, respectively (Supplemental Table 1). The 9th generation airways had relatively thinner wall than the 6th generation (Supplemental Table 2). For both generation airways, BMI was significantly associated with increased wall areas and age was significantly associated with increased lumen areas (Supplemental Table 3 and 4). Consistent with our previous findings in carbon black study [21], lobar diversity was identified for airway dimensions with RB1 having thinner wall, narrower lumen, and smaller airway compared to other three airways of 6th generation regardless of DE exposure status (Supplemental Table 1 and 3). For the 9th generation airways, the airways of lower lobes (i.e., LB9 and RB9) had thicker airway wall, wider lumen, and larger airway compared to airways of upper lobes regardless of DE exposure status (i.e., LB1 + 2 and RB1, Supplemental Table 1 and 4). Interestingly, cigarette smoking status and packyears had no effects on any of these measurements (Supplemental Table 3 and 4).

### Diesel exhaust exposure and airway dimensions

Linear mixed effects (LME) model identified higher wall and lumen areas and larger airways in DETs versus non-DETs for both 6th and 9th generations of airways (Supplemental Table 5 and 6). However, significant associations were only identified for wall area of 6th generation of airways and for airway area of both generations of airways. Moreover, significantly increased wall area percent was identified in DETs versus non-DETs for 6th generation of airways (*P* = 0.031), but not for 9th generation of airways (*P* = 0.74, Table 2). We also assessed self-reported dust exposure in their prior employment history, and a total of 12 DETs and 2 non-DETs reported ever dust exposure in their previous jobs. The difference of wall area percent of 6th generation of airways between the two study groups became more significant (ratio = 1.07, 95%CI = 1.01–1.14, *P* = 0.026) when these 14 subjects were excluded. The difference of wall area percent of 9th generation of airways remained non-significant (*P* = 0.76). In addition, no significant interaction was identified between cigarette smoking status and DE exposure on wall area percent of 6th generation of airways (*P* = 0.86). Moreover, the effect of DE exposure on wall area percent of 6th generation of airways did not vary by lung lobe (*P* = 0.77) as well.

**Table 2:** The effect of diesel exhaust exposure on wall area percent in all study subjects (*n* = 154)^a^

Variable	Non-DET (n = 76)	DET (n = 78)	Ratio (95CI%)	*P*
6th wall area percent (%)				
LB1 + 2	54.1 (45.5, 63.9)	57.4 (50.3, 64.8)		
LB9	51.1 (42.8, 59.4)	56.0 (48.2, 62.8)		
RB9	55.3 (46.2, 61.3)	56.4 (49.5, 63.5)		
RB1	53.9 (43.7, 63.6)	57.0 (50.8, 64.3)		
All	52.0 (49.9, 54.3)	55.4 (53.0, 58.0)	1.07 (1.01, 1.13)	0.031
9th wall area percent (%)				
LB1 + 2	41.6 (34.0, 51.4)	45.1 (35.4, 54.4)		
LB9	43.6 (29.9, 54.7)	45.1 (33.6, 55.0)		
RB9	46.5 (33.8, 56.4)	42.2 (31.0, 56.7)		
RB1	45.8 (37.2, 58.9)	46.6 (38.6, 57.6)		
All	43.6 (40.7, 46.6)	44.2 (41.2, 47.5)	1.02 (0.93, 1.11)	0.744

Definition of abbreviations: *CI* confidence interval, *DET* diesel engine testers, *LB* left bronchus, *RB* right bronchus

^a^ Linear mixed effects model assessed differences of natural log transformed 6th and 9th wall area percent between non-DETs and DETs with adjustment of age, BMI, smoking history, CT reconstruction method, and lung lobes. Indices were shown as median (Q1, Q3). Ratio = e^β^, 95%CI = e^(β ± 1.96 × Se)^. Descriptive statistics for all were exponentials of least square means and 95%CIs that were calculated based on natural log transformed data using linear mixed effects model with adjustment for covariates listed above and may be regarded as an overall level of wall area percent based on the four sampled airways of 6th or 9th generations

A total of 53 non-DETs and 53 DETs with CT imaging data had CCAM available [13]. Study subjects were divided equally into 5 groups based on CCAM levels to characterize the dose-response relationship between DE exposure and wall area percent (Table 3). Interestingly, although subjects with CCAM greater than 1.33% tend to have increased levels of wall area percent of 6th generation of airways, only subjects in the highest group of CCAM (≥4.7%) had significantly increased levels (*P* = 0.015). The overall trend test was of borderline significance (*P* = 0.063). A similar does-dependent pattern was observed for 9th generation of airway as well, though the association was not statistically significant.

**Table 3:** The dose-response between CCAM and wall area percent in all study subjects (*n* = 106)^a^

Wall area percent	CCAM		N (DET)	Mean (95%CI)	Ratio (95%CI)	*P*
	Category	Range (%)				
6th generation (%)	1	0.24–1.33	21 (2)	49.7 (45.9, 53.7)	Ref	
	2	1.33–1.92	21 (4)	54.3 (50.1, 58.9)	1.09 (0.98, 1.22)	0.102
	3	1.92–3.10	21 (9)	53.0 (49.0, 57.1)	1.07 (0.96, 1.19)	0.249
	4	3.10–4.70	21 (18)	52.0 (48.1, 56.3)	1.05 (0.94, 1.17)	0.391
	5	4.70–30.24	22 (20)	56.8 (52.5, 61.3)	1.14 (1.03, 1.27)	0.015
Trend test^b^					1.02 (1.00, 1.05)	0.063
9th generation (%)	1	0.24–1.33	21 (2)	39.4 (35.1, 44.3)	Ref	
	2	1.33–1.92	21 (4)	45.3 (40.2, 51.1)	1.15 (0.98, 1.35)	0.085
	3	1.92–3.10	21 (9)	43.9 (39.2, 49.1)	1.11 (0.95, 1.31)	0.195
	4	3.10–4.70	21 (18)	43.0 (38.3, 48.3)	1.09 (0.93, 1.28)	0.287
	5	4.70–30.24	22 (20)	45.1 (40.2, 50.6)	1.14 (0.98, 1.34)	0.098
Trend test^b^					1.02 (0.99, 1.06)	0.239

Definition of abbreviations: *CCAM* carbon content in airway macrophage, *CI* confidence interval, *DET* diesel engine tester, *LsMean* least square mean, *Ref* reference

^a^ Linear mixed effects model assessed differences of natural log transformed 6th and 9th wall area percent between categories 2–5 and the reference group (category 1) with adjustment of age, BMI, smoking history, CT reconstruction method, and lung lobes. Mean and 95%CI are exponentials of least square means and 95%CIs calculated based on natural log transformed data. Ratio = e^β^, 95%CI = e^(β ± 1.96 × Se)^

^b^ Linear mixed effects model assessed dose – response relation between CCAM category coded as 0, 1, 2, 3, and 4 and natural log transformed 6th and 9th wall area percent with adjustment for covariates listed above

### Correlations between wall area percent and spirometry

**Fig. 2:**
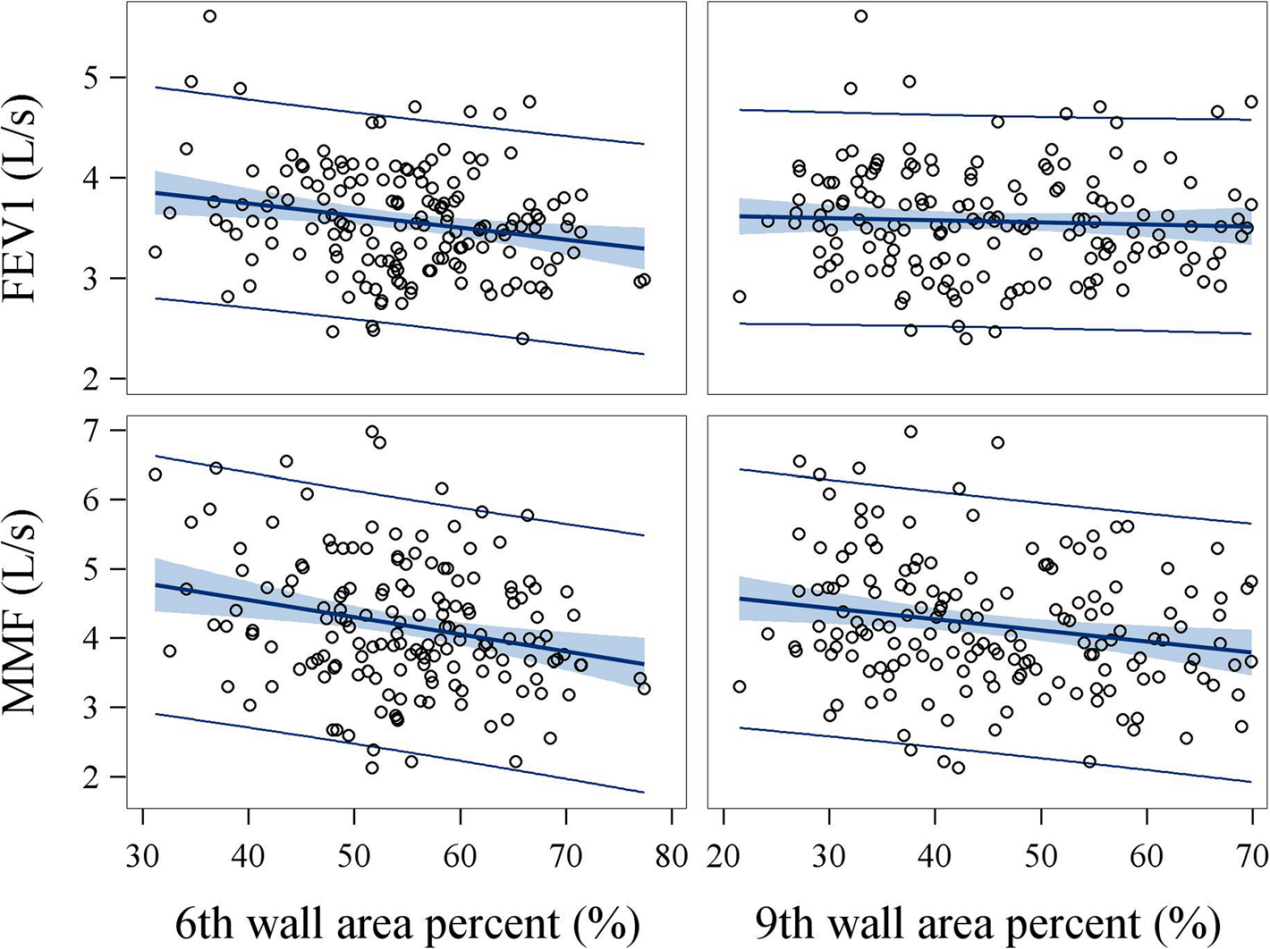
Correlation between wall area percent and spirometry. Values of wall area percent from four airways were averaged and assessed for their correlations with spirometry. Highly significant inverse correlations were identified between wall area percent and MMF. Pearson coefficients for correlations of 6th wall area percent with FEV1 and MMF were − 0.24 (*P* = 0.0028) and − 0.27 (*P* = 0.0008), respectively. Pearson coefficients for correlations of 9th wall area percent with FEV1 and MMF were − 0.06 (*P* = 0.47) and − 0.24 (P = 0.0028), respectively. FEV1 = forced expiratory volume in 1 s; MMF = maximal mid-expiratory flow

We averaged wall area percent from four airways to assess their association with spirometry as the outcome using generalized linear model (Table 4). Wall area percent was inversely associated with FEV1, FEV1/FVC ratio, maximal mid expiratory flow (MMF), FEF50, and FEF75. As the very beginning segments of small airways, wall area percent of 6th and 9th generations of airways were more significantly associated with MMF, FEF50, and FEF75 than with FEV1 and FEF25 with MMF having the most significant associations for both generations of airways (Fig. 2). Each 1% increase of wall area percent at 6th or 9th generations of airways was associated with a reduction of 31 ml/s and 22 ml/s in MMF (all Ps < 0.001), respectively.

**Table 4:** Association between wall area percent and spirometry in all study subjects (n = 154)^a^

Wall area percent	Spirometry	β	SE	*P*
6th generation (%)	FEV1 (L/S)	−0.011	0.004	0.005
	FVC (L)	−0.005	0.004	0.254
	FEV1/FVC (%)	−0.147	0.050	0.004
	MMF (L/S)	−0.031	0.008	< 0.001
	PEF (L/S)	−0.005	0.013	0.709
	FEF25 (L/s)	−0.018	0.013	0.168
	FEF50 (L/s)	−0.033	0.011	0.003
	FEF75 (L/s)	−0.024	0.006	< 0.001
9th generation (%)	FEV1 (L/S)	−0.006	0.003	0.040
	FVC (L)	−0.001	0.003	0.754
	FEV1/FVC (%)	−0.110	0.036	0.003
	MMF (L/S)	−0.022	0.006	< 0.001
	PEF (L/S)	−0.005	0.009	0.639
	FEF25 (L/s)	−0.016	0.009	0.094
	FEF50 (L/s)	−0.025	0.008	0.002
	FEF75 (L/s)	−0.015	0.005	0.001

Definition of abbreviations: *FEF25* Forced expiratory flow rate at 25% vital capacity, *FEF50* Forced expiratory flow rate at 50% vital capacity, *FEF75* Forced expiratory flow rate at 75% vital capacity, *FEV1* forced expiratory volume in 1 s, *FVC* forced vital capacity, *L/S* liters per second, *MMF* maximal mid-expiratory flow, *PEF* peak expiratory flow, *SE* standard error

^a^ Generalized linear model assessed the associations of spirometry as outcome with average wall area percent with adjustment of age, height, BMI, smoking history. β is calculated per 1% increase in average wall area percent

### Mediational effects of wall area percent on associations between DE exposure and FEV1

We tested the hypothesis whether the effect of DE exposure on FEV1 could be mediated by airway wall thickening. Mediation analyses identified that 20% of association between DE exposure status and FEV1 could be explained by the inclusion of wall area percent of 6th generation of airways in the model (P_perm_ = 0.028, Supplemental Table 7).

## Discussion

With the 64-slice quantitative CT and advanced analytical informatics platform, we were able to obtain reliable measurements of dimensions for 6th and 9th generations of airways, the very beginning segments of small airways, in workers exposed to a high level of DE aerosol. More significant correlations of wall area percent at either generations with small airway function (i.e., MMF, FEF50, and FEF75 [34]) compared to the correlations with larger airway function (i.e., FEV1 and FEF25) were identified, supporting that airway dimensions at 6th and 9th generations of airways may be more relevant to small or distal airway function. Considering none of the study subjects met the spirometry diagnosis of airway obstruction (i.e., FEV1/FVC < 70%), wall thickening of 6th generation airways without lumen narrowing may be an early pathological change for pulmonary toxicity of the chronic DE exposure. Airways in all lobes were similarly affected, suggesting a relatively even distribution of particulates deposited in different parts of the lung. Most importantly, mediation analysis identified that FEV1 reduction due to DE exposure could be partially explained by wall thickening of the 6th generation of airways, supporting the importance of airway morphology in affecting lung function measurements [35, 36].

Small airways refer to those airways around 2 mm and less in diameter and generally include generations from 8th to 19th [37]. Our measurements confirmed that airways of 6th and 9th generations as the very beginning segments of small airways had diameters of 4.4–5.0 mm and 2.6–2.9 mm, respectively. Due to the narrow lumen, no cartilage in the airway wall, and lack of mucociliary transfer system, small airways represent the major site of particle deposition in fine particulate matter exposed population and airflow limitation in patients with chronic lung diseases. Spirometry is not sensitive to small airway diseases because small airways only contribute a little to airflow resistance (10–25%), and obstruction of a large percentage of all small airways is required to show any changes in FEV1 measures [37]. In this case, high resolution CT acquired at end-inhalation and advanced informatics platform make it possible for precise quantification of airway dimensions for small airways, at least for the beginning segments of small airways. Thus, our assessment may monitor the degree of small airway remodeling in particulate matter exposed populations prior to the detection of compromised lung spirometry (e.g., FEV1/FVC < 70%). Detection of air trapping in end-inhalation CT has been proposed as an imaging CT feature for small airway diseases in COPD patients [37]. However, based on the young age of the study populations and the healthy lung function (e.g., average FEV1 percent predicted over 94%), we do not expect that the lungs of DETs have been damaged that much. However, we plan to include end-inhalation CT in our future follow-up visits for these DETs and will include older or retired DETs to characterize the temporal change of lung tissue remodeling including air trapping. In addition, oscillometry which is effort-independent and sensitive to small airway changes will be included in addition to spirometry in our future studies of particulate matter exposure [38].

The lower packyears in ever smokers in DETs could be attributed to the non-smoking rule within the company properties. This pattern is also seen in our published study that carbon black packers had higher smoking rate however with lower packyears in ever smokers compared to non-carbon black packer controls [21]. We took a very careful approach to handle cigarette smoking in this study which included stratification analysis by smoking status, smoking and DE exposure interaction analyses, and adjustment of smoking history in the models. We found no influence of smoking status on macrophage carbon load and airway dimensions of either generations of airways. The effect of DE exposure on airway dimensions did not vary by smoking status. Thus, even the smoking history was not completely balanced between the two study groups, we do not expect any confounding effects of cigarette smoking on our main associations identified.

Accumulating studies have supported wall area percent of segmental or subsegmental airways or estimated mean square root wall area of 10-mm internal perimeter airways as a CT imaging biomarker associated with former occupational inorganic [39, 40] or organic [41] dust exposure or current cigarette smoking [35, 42] in senior people. Our most recent study of carbon black packers also identified a linear dose-response relationship between CCAM and wall area percent of 6th and 9th generations of airways [21]. Additional analyses found thicker airway wall and narrower lumen with no change in total airway size in carbon black packers compared to non-carbon black packers [21]. Thus, airway wall thickening was the major driver for the observed elevation of wall area percent in carbon black packers and animal studies suggested that this may result from hyperplastic change of bronchus epithelium [43, 44]. Interestingly, despite elevated wall area percent and wall area in DETs compared to non-DETs, lumen narrowing was not observed, suggesting that the pathological basis for airway wall thickening induced by chronic DE exposure may be different to that for carbon black exposure. The lack of lumen narrowing in the lungs of DETs may also explain the modest mediation effect size (20%) of 6th wall area percent on the association between DE exposure status and FEV1 seen in this study in comparison to an almost complete mediation (72%) seen in the carbon black packer study [21]. Carbon black has been regarded as a carbonaceous core analog for many airborne soot-containing particulate matters including DE particulates. Our own analysis of carbon black and DE particulates collected from the field and others identified considerable similarity in primary sphere diameter, high surface area per mass values, and aciniform morphology, though DE particulates tend to form smaller aggregates and agglomerates and contain high percentage of organic carbon whereas carbon black is almost pure elemental carbon [13, 14, 45, 46]. Thus, the different patterns in airway dimensional changes between carbon black packers and DETs may rooted from the constituents that carbonaceous cores of DE particulates carry and volatile gases in DE. Post mortem studies of lung tissues from Mexico City and Vancouver residents had identified that chronic exposure to high levels of particulate air pollution resulted in presence of carbonaceous aggregates of ultrafine particles in the airway mucosa and abnormal small airways with fibrotic walls and excess muscle [47]. Diesel emission particle is a major source of inhalable particulate matters in ambient air in many urban areas. Ambient particulate matters have similar composition as to diesel emission particles. Thus, we expect similar pathological changes may occur in small airways exposed to diesel emission particulates which may explain the thickened airway walls observed in DETs versus non-DETs under CT assessment. Nevertheless, future studies based on lung biopsies (e.g., bronchoscopy brushing) in exposed workers may delineate airway wall components contributing to small airway wall thickening observed under quantitative CT.

The principal strength of this study derived from a unique DET cohort that has been thoroughly characterized for its particulate matter exposure over time and has very little co-exposures (such as mine dust, gasoline emission, and silica dust) that may confound the effects of diesel emission exposure [13, 14]. Second, the application of a valid and improved CCAM methodology with better precision minimizes the possibility of technical artifacts for processing sputum samples from cigarette smokers and also avoids biases derived from different size of macrophages and carbon particles overlapping with nuclei [13, 26, 48]. Third, with the 64-slice quantitative CT and advanced analytical informatics platform, we were able to reliably and directly measure dimensions of multiple smaller airways.

Radiological reports based on end-inspiration CT did not identify evidence of increased prevalence of lung abnormality in DETs and no workers have met spirometry diagnosis of airway obstruction (i.e., FEV1/FVC < 0.70) yet. This suggested that in this young group of study subjects, small airway wall thickening without lumen narrowing could be an early pathological event contributing to lung injury caused by chronic exposure to DE and emerges prior to the occurrence of large airway diseases or emphysematous or fibrotic changes. However, as a cross-sectional study, the contribution of small airway wall thickening to DE exposure induced chronic lung diseases such as COPD and the reversibility of observed changes could not be delineated.

## Conclusions

With the maturation of quantitative assessment of airway morphology and better resolution, quantitative CT could specifically assess the dimensional alterations of small airways prior to the emergence of compromised lung spirometry and may also provide toxicological and pathological hints of lung injury through comparative studies across various particulate matter exposures. Our study identified imaging evidence supporting small airway wall thickening without lumen narrowing as potential early pathological changes in the lungs of DETs. The differential patterns of changes in small airway dimensions between DE and carbon black aerosol exposures suggest constituents other than carbon cores in DE may contribute to such differences. Our findings contribute to the understanding of the pulmonary toxicity caused by many airborne soot-containing particulate matters and may also assist disentangling the toxicity and health effects of different PM constituents through making comparisons to airway remodeling seen in carbon black packers [21, 45, 49, 50].

## Materials and methods

### Study subjects

DET cohort was established in 2012 with detailed description of inclusion and exclusion criteria, working environment, and environmental monitoring introduced in references [12, 15]. The DET group included 137 male workers from a diesel engine manufacturing plant who had been testing heavy-duty diesel engines for at least six months prior to the enrollment into this study. The non-DETs group consisted of 127 male water pump management workers from a local water utility company residing in the same city as the diesel engine testing workshops. Follow-up of the DET cohort was conducted in 2018 and enrolled 90 DETs and 88 non-DETs, among which 51 DETs and 55 non-DETs were also studied in 2012. The new enrollment followed the same inclusion and exclusion criteria as the 2012 study [51]. In the current study, five subjects with missing smoking history only, 17 subjects not receiving end-inhalation lung CT scanning only, and two subjects missing both were excluded and this led to a final sample size of 78 DETs and 76 non-DETs (Table 1). Written informed consent was acquired from all participants prior to the interview and any procedures. The protocol was approved by the Medical Ethical Review Committee of the National Institute for Occupational Health and Poison Control, Chinese Center for Disease Control and Prevention (protocol number: NIOHP201604).

### Environmental exposure assessment

Fine particulate matter (PM2.5) levels, PM2.5 related EC, organic carbon (OC), and total carbon (TC) inside the DET work plants and in reference areas had been assessed four times since 2012 with the most recent assessment conducted during the field visit of this study in 2018 and were summarized in reference [13] with detailed method described in reference [14]. The reference areas were within the water utility company, were about seven kilometers away from the diesel engine testing workshops, and were away from the main roads with heavy traffic. Results provided in Table 1 were collected from the 2018 field visit. In addition, the particulate size distribution, PM2.5 related elemental carbon, organic carbon, and total carbon, non-carcinogenic (*n* = 8) and carcinogenic (n = 8) PAHs in the particle phase, and NO2 and SO2 in the gas phase sampled inside the diesel engine testing workshops and the reference areas were thoroughly profiled in the 2014 field visit [14]. Moreover, urinary metabolites of several PAHs were also assessed in the 2012 study and confirmed the much higher exposure of polycyclic aromatic hydrocarbons in DETs versus non-DETs [15].

### CCAM assay

Sputum collection, processing, slide preparation, and quality assessment were described in details in reference [13]. For each study subject, randomly selected and well-stained macrophages (*n* = 50) with intact cytoplasm and clear nucleus staining were captured to calculating the proportion of cytoplasm area occupied by carbon particles with upper quartile defined as the CCAM index to quantify the bio-effective dose of EC exposure in the lungs and for all association analyses. The utility of the proportion of nucleus negative cytoplasm area occupied by carbon particles may avoid biases derived from different size of macrophages due to cigarette smoking and DE exposure and carbon particles overlapping with nuclei [13, 26, 48].

### Quantitative CT assessment of small airway dimensions

**Fig. 3:**
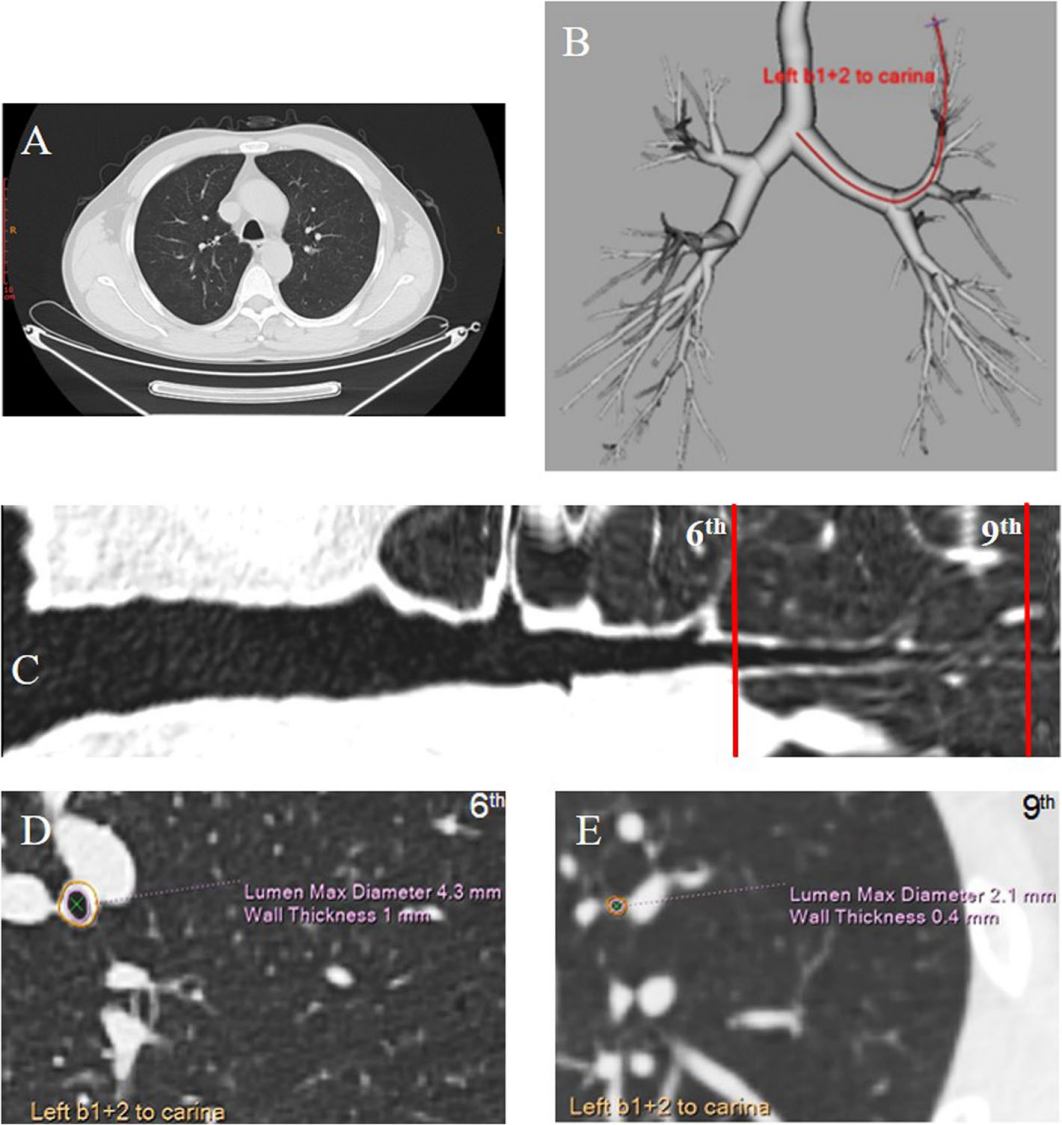
Reconstruction of tracheobronchial tree and measurement of airway dimensions. One axial image of chest CT scan (**a**) and reconstructed image of tracheobronchial tree (**b**) for a study subject were presented with the candidate airway for measurement highlighted by the red line. The airway straightened was shown in C with the measurement spots for the 6th and 9th generations labeled by the red lines. The cross-sectional images of airways for dimensional measurement were presented in D (6th generation) and E (9th generation) with lumen diameter and wall thickness labeled

CT scans at full inspiration were acquired using a 64-slice CT scanner (OPTIMA CT660, GE Healthcare, USA) from apex to base of the lung without contrast. The scanning parameters were set at 120 kVp, auto mA, gantry rotation of 0.6 r/s, pitch of 0.985, 0.625 mm slice thickness, and continuous slices. The standard or lung reconstruction kernel was used to construct the images. The board-certified radiologist (Dr. Qianli Ma) did not participate the field survey and did not know the exposure status of the study subjects when making radiographic diagnosis. Detailed procedure of airway measurement was described in reference [21]. Three-dimension reconstruction of lungs and airway trees were conducted using the Philips IntelliSpace Portal 9.0 (Royal Philips, Netherlands). Four broncho-pulmonary segmental airways including right bronchi 1 (i.e., apical segment, RB1) and 9 (i.e., lateral basal segment, RB9) and left bronchi 1 + 2 (i.e., apicoposterior segment, LB1 + 2) and 9 (i.e., lateral basal segment, LB9) were selected as they run orthogonal to the axial lane offering more accurate measurements. The 6th generation was chosen as a most popularly studied subsegment [37], while the 9th generation was chosen because it is the most distant airway that can be reliably visualized and measured using the Philips IntelliSpace Portal 9.0. A well-trained analyst manually highlighted the four candidate broncho-pulmonary segments (i.e., RB1, RB9, LB1 + 2, and LB9) and carefully scrutinized along the 3-D airway path towards the diagonal peripheral of corresponding lung lobes to localize the longest bronchi-bronchiole that was visible for each selected segmental bronchus (Fig. 3). The visualization platform allows for a free rotating of the airway which facilitates an all-angle inspection of the airway branches. Crude airway dimensional measurements (e.g., wall area, lumen area, and airway area) were obtained by the software at the midpoint between the parent and the daughter branch points that was manually labelled by the analyst [21].

### Spirometry

Spirometry was conducted without inhaling bronchodilator by a certified technician using a portable calibrated electronic spirometer (CHESTGRAPH HI-701, Japan) in accordance with the American Thoracic Society and European Respiratory Society standards [52].

### Statistical analysis

The Wilcoxon rank sum test was used to compare the differences in CCAM by smoking or DE exposure status. Due to inter-lobe variation of airway dimensions (e.g., wall, lumen, and airway areas and wall area percent) across the four studied airways (Supplemental Table 1 and 2), we regarded values from four airways as repeated measurements and modeled their associations with DE exposure and other variables such as lung lobe and demographics using linear mixed effects (LME) model. Age, body mass index (BMI), smoking history (i.e., smoking status and packyears), CT reconstruction method (i.e., standard versus lung), and lung lobes were selected a priori as covariates for adjustment for association analyses with airway dimensions as the outcome. Two interaction analyses including lung lobes × DE exposure and cigarette smoking status × DE exposure were designed a priori. Age, height, BMI, and smoking history were selected a priori as covariates for adjustment in models with spirometry as the outcome. In addition to binary DE exposure status, CCAM was also used as a bio-effective dosimeter for exposure to carbon black aerosol in the lungs to explore the dose-response relationship in which analysis all study subjects were evenly divided into five groups based on the CCAM levels. All airway measurements were natural log transformed to improve normality of the residual and satisfy the homoscedasticity assumption. Mediation analysis with permutation-based statistics [13, 21] was conducted to assess whether DE exposure status and spirometry associations could be mediated by inclusion of wall area percent. All statistical analyses were conducted using SAS (version 9.4, NC, USA, site 70,239,492).

## Supplementary Information

**Additional file 1: Supplemental Table 1**. The anatomical diversity of airway measurements assessed in non-DETs and DETs separately with airway LB1 + 2 as the reference (*n* = 154)^a^.

**Additional file 2: Supplemental Table 2**. The anatomical diversity of wall area percent assessed in non-DETs and DETs separately with airway LB1 + 2 as the reference (n = 154)^a^.

**Additional file 3: Supplemental Table 3**. The influencing factors for 6th generation airway dimensions in all study subjects (n = 154)^a^.

**Additional file 4: Supplemental Table 4**. The influencing factors for 9th generation airway dimensions in all study subjects (n = 154)^a^.

**Additional file 5: Supplemental Table 5.** The effect of diesel exhaust exposure status on 6th generation airway dimensions in all study subjects (n = 154)^a^.

**Additional file 6: Supplemental Table 6**. The effect of diesel exhaust exposure status on 9th generation airway dimensions in all study subjects (n = 154)^a^.

**Additional file 7: Supplemental Table 7**. Mediation effect of airway wall thickening (measured by wall area percent) on associations between diesel exhaust exposure and FEV1 in all subjects (n = 154)^a^.

## Data Availability

Detailed quantification pipeline of airway dimensions, CT imagines, SAS codes, and datasets analyzed during the current study are available from the corresponding author on reasonable request.

## References

[1] Benbrahim-Tallaa L, Baan RA, Grosse Y, Lauby-Secretan B, El Ghissassi F, Bouvard V (2012). Carcinogenicity of diesel-engine and gasoline-engine exhausts and some nitroarenes. Lancet Oncol.

[2] United States (2002). Environmental Protection Agency. Office of Research and Development., National Center for environmental assessment (Washington D.C.): health assessment document for diesel engine exhaust.

[3] Eastwood P (2008). Particulate emissions from vehicles. Chichester, England.

[4] Breton CV, Salam MT, Wang X, Byun HM, Siegmund KD, Gilliland FD (2012). Particulate matter, DNA methylation in nitric oxide synthase, and childhood respiratory disease. Environ Health Perspect.

[5] Ristovski ZD, Miljevic B, Surawski NC, Morawska L, Fong KM, Goh F (2012). Respiratory health effects of diesel particulate matter. Respirology..

[6] Steiner S, Bisig C, Petri-Fink A, Rothen-Rutishauser B (2016). Diesel exhaust: current knowledge of adverse effects and underlying cellular mechanisms. Arch Toxicol.

[7] Rudell B, Ledin MC, Hammarstrom U, Stjernberg N, Lundback B, Sandstrom T (1996). Effects on symptoms and lung function in humans experimentally exposed to diesel exhaust. Occup Environ Med.

[8] Andersen MHG, Frederiksen M, Saber AT, Wils RS, Fonseca AS, Koponen IK (2019). Health effects of exposure to diesel exhaust in diesel-powered trains. Part Fibre Toxicol.

[9] Du M, Hall GL, Franklin P, Musk AB, Mullins BJ, de Klerk N (2020). Association between diesel engine exhaust exposure and lung function in Australian gold miners. Int J Hyg Environ Health.

[10] Ulvestad B, Lund MB, Bakke B, Thomassen Y, Ellingsen DG (2015). Short-term lung function decline in tunnel construction workers. Occup Environ Med.

[11] Lotz G, Plitzko S, Gierke E, Tittelbach U, Kersten N, Schneider WD (2008). Dose-response relationships between occupational exposure to potash, diesel exhaust and nitrogen oxides and lung function: cross-sectional and longitudinal study in two salt mines. Int Arch Occup Environ Health.

[12] Zhang X, Duan H, Gao F, Li Y, Huang C, Niu Y, Gao W, Yu S, Zheng Y (2015). Increased micronucleus, nucleoplasmic bridge, and nuclear bud frequencies in the peripheral blood lymphocytes of diesel engine exhaust-exposed workers. Toxicol Sci.

[13] Cheng W, Liu Y, Tang J, Duan H, Wei X, Zhang X, Yu S, Campen MJ, Han W, Rothman N, Belinsky SA, Lan Q, Zheng Y, Leng S (2020). Carbon content in airway macrophages and genomic instability in Chinese carbon black packers. Arch Toxicol.

[14] Niu Y, Zhang X, Meng T, Wang H, Bin P, Shen M, Chen W, Yu S, Leng S, Zheng Y (2018). Exposure characterization and estimation of benchmark dose for cancer biomarkers in an occupational cohort of diesel engine testers. J Expo Sci Environ Epidemiol.

[15] Wang H, Duan H, Meng T, Yang M, Cui L, Bin P, Dai Y, Niu Y, Shen M, Zhang L, Zheng Y, Leng S (2018). Local and systemic inflammation may mediate diesel engine exhaust-induced lung function impairment in a Chinese occupational cohort. Toxicol Sci.

[16] Dournes G, Laurent F (2012). Airway Remodelling in asthma and COPD: findings, similarities, and differences using quantitative CT. Pulmon Med.

[17] Bergeron C, Tulic MK, Hamid Q (2007). Tools used to measure airway remodelling in research. Eur Respir J.

[18] Fehrenbach H, Wagner C, Wegmann M (2017). Airway remodeling in asthma: what really matters. Cell Tissue Res.

[19] Petersen H, Vazquez Guillamet R, Meek P, Sood A, Tesfaigzi Y (2018). Early Endotyping: a chance for intervention in chronic obstructive pulmonary disease. Am J Respir Cell Mol Biol.

[20] Kirby M, Tanabe N, Tan WC, Zhou G, Obeidat M, Hague CJ (2018). Total Airway Count on Computed Tomography and the Risk of Chronic Obstructive Pulmonary Disease Progression. Findings from a Population-based Study. Am J Respir Crit Care Med.

[21] Cao X, Lin L, Sood A, Ma Q, Zhang X, Liu Y, et al. Small airway wall thickening assessed by computerized tomography is associated with low lung function in Chinese carbon black packers. Toxicol Sci. 2020; in press. 10.1093/toxsci/kfaa134 PMC7825005 32818265

[22] Ge C, Peters S, Olsson A, Portengen L, Schuz J, Almansa J (2020). Diesel Engine Exhaust Exposure, Smoking, and Lung Cancer Subtype Risks. A Pooled Exposure-Response Analysis of 14 Case-Control Studies. Am J Respir Crit Care Med.

[23] Bai Y, Casas L, Scheers H, Janssen BG, Nemery B, Nawrot TS (2018). Mitochondrial DNA content in blood and carbon load in airway macrophages. A panel study in elderly subjects. Environ Int.

[24] Jacobs L, Emmerechts J, Hoylaerts MF, Mathieu C, Hoet PH, Nemery B (2011). Traffic air pollution and oxidized LDL. PLoS One.

[25] Jacobs L, Emmerechts J, Mathieu C, Hoylaerts MF, Fierens F, Hoet PH, Nemery B, Nawrot TS (2010). Air pollution related prothrombotic changes in persons with diabetes. Environ Health Perspect.

[26] Bai Y, Brugha RE, Jacobs L, Grigg J, Nawrot TS, Nemery B (2015). Carbon loading in airway macrophages as a biomarker for individual exposure to particulate matter air pollution - a critical review. Environ Int.

[27] Nwokoro C, Ewin C, Harrison C, Ibrahim M, Dundas I, Dickson I, Mushtaq N, Grigg J (2012). Cycling to work in London and inhaled dose of black carbon. Eur Respir J.

[28] Belli AJ, Bose S, Aggarwal N, DaSilva C, Thapa S, Grammer L, Paulin LM, Hansel NN (2016). Indoor particulate matter exposure is associated with increased black carbon content in airway macrophages of former smokers with COPD. Environ Res.

[29] Whitehouse AL, Miyashita L, Liu NM, Lesosky M, Flitz G, Ndamala C, Balmes JR, Gordon SB, Mortimer K, Grigg J (2018). Use of cleaner-burning biomass stoves and airway macrophage black carbon in Malawian women. Sci Total Environ.

[30] Kulkarni N, Pierse N, Rushton L, Grigg J (2006). Carbon in airway macrophages and lung function in children. N Engl J Med.

[31] Kalappanavar NK, Vinodkumar CS, Gouli C, Sanjay D, Nagendra K, Basavarajappa KG, Patil R (2012). Carbon particles in airway macrophage as a surrogate marker in the early detection of lung diseases. Int J Occup Environ Med.

[32] Dai Y, Zhang X, Zhang R, Zhao X, Duan H, Niu Y, Huang C, Meng T, Ye M, Bin P, Shen M, Jia X, Wang H, Yu S, Zheng Y (2016). Long-term exposure to diesel engine exhaust affects cytokine expression among occupational population. Toxicol Res (Camb).

[33] Müller NJC. CT diagnosis of emphysema. It may be accurate, but is it relevant? 1993;103, 2:329–30. 10.1378/chest.103.2.329. 10.1378/chest.103.2.329 8432110

[34] Stockley JA, Cooper BG, Stockley RA, Sapey E (2017). Small airways disease: time for a revisit?. Int J Chron Obstruct Pulmon Dis.

[35] Washko GR, Diaz AA, Kim V, Barr RG, Dransfield MT, Schroeder J (2014). Computed tomographic measures of airway morphology in smokers and never-smoking normals. J Appl Physiol (1985).

[36] Diaz AA, Rahaghi FN, Ross JC, Harmouche R, Tschirren J, San Jose Estepar R (2015). Understanding the contribution of native tracheobronchial structure to lung function: CT assessment of airway morphology in never smokers. Respir Res.

[37] McNulty W, Usmani OS. Techniques of assessing small airways dysfunction. Eur Clin Respir J. 2014;1(1). 10.3402/ecrj.v1.25898. https://www.ncbi.nlm.nih.gov/pubmed/26557240. 10.3402/ecrj.v1.25898 PMC4629724 26557240

[38] Lundblad LKA, Siddiqui S, Bossé Y, Dandurand RJ (2019). Applications of oscillometry in clinical research and practice. Can J Respir Crit Care Sleep Med.

[39] Paulin LM, Smith BM, Koch A, Han M, Hoffman EA, Martinez C, Ejike C, Blanc PD, Rous J, Barr RG, Peters SP, Paine R, Pirozzi C, Cooper CB, Dransfield MT, Comellas AP, Kanner RE, Drummond MB, Putcha N, Hansel NN (2018). Occupational exposures and computed tomographic imaging characteristics in the SPIROMICS cohort. Ann Am Thorac Soc.

[40] Marchetti N, Garshick E, Kinney GL, McKenzie A, Stinson D, Lutz SM, Lynch DA, Criner GJ, Silverman EK, Crapo JD, COPDGene Investigators (2014). Association between occupational exposure and lung function, respiratory symptoms, and high-resolution computed tomography imaging in COPDGene. Am J Respir Crit Care Med.

[41] Lai PS, Hang JQ, Zhang FY, Sun J, Zheng BY, Su L, Washko GR, Christiani DC (2016). Imaging phenotype of occupational endotoxin-related lung function decline. Environ Health Perspect.

[42] Donohue KM, Hoffman EA, Baumhauer H, Guo J, Budoff M, Austin JH (2012). Cigarette smoking and airway wall thickness on CT scan in a multi-ethnic cohort: the MESA lung study. Respir Med.

[43] Chu C, Zhou L, Xie H, Pei Z, Zhang M, Wu M, Zhang S, Wang L, Zhao C, Shi L, Zhang N, Niu Y, Zheng Y, Zhang R (2019). Pulmonary toxicities from a 90-day chronic inhalation study with carbon black nanoparticles in rats related to the systemical immune effects. Int J Nanomedicine.

[44] Han B, Chu C, Su X, Zhang N, Zhou L, Zhang M, Yang S, Shi L, Zhao B, Niu Y, Zhang R (2020). N(6)-methyladenosine-dependent primary microRNA-126 processing activated PI3K-AKT-mTOR pathway drove the development of pulmonary fibrosis induced by nanoscale carbon black particles in rats. Nanotoxicology..

[45] Long CM, Nascarella MA, Valberg PA (2013). Carbon black vs. black carbon and other airborne materials containing elemental carbon: physical and chemical distinctions. Environ Pollut.

[46] Dai Y, Niu Y, Duan H, Bassig BA, Ye M, Zhang X, Meng T, Bin P, Jia X, Shen M, Zhang R, Hu W, Yang X, Vermeulen R, Silverman D, Rothman N, Lan Q, Yu S, Zheng Y (2016). Effects of occupational exposure to carbon black on peripheral white blood cell counts and lymphocyte subsets. Environ Mol Mutagen.

[47] Churg A, Brauer M, del Carmen A-CM, Fortoul TI, Wright JL (2003). Chronic exposure to high levels of particulate air pollution and small airway remodeling. Environ Health Perspect.

[48] Frankenberger M, Menzel M, Betz R, Kassner G, Weber N, Kohlhaufl M (2004). Characterization of a population of small macrophages in induced sputum of patients with chronic obstructive pulmonary disease and healthy volunteers. Clin Exp Immunol.

[49] Stoeger T, Reinhard C, Takenaka S, Schroeppel A, Karg E, Ritter B, Heyder J, Schulz H (2006). Instillation of six different ultrafine carbon particles indicates a surface area threshold dose for acute lung inflammation in mice. Environ Health Perspect.

[50] Swafford DS, Nikula KJ, Mitchell CE, Belinsky SA (1995). Low frequency of alterations in p53, K-ras, and mdm2 in rat lung neoplasms induced by diesel exhaust or carbon black. Carcinogenesis..

[51] Bin P, Shen M, Li H, Sun X, Niu Y, Meng T, Yu T, Zhang X, Dai Y, Gao W, Gu G, Yu S, Zheng Y (2016). Increased levels of urinary biomarkers of lipid peroxidation products among workers occupationally exposed to diesel engine exhaust. Free Radic Res.

[52] Miller MR, Hankinson J, Brusasco V, Burgos F, Casaburi R, Coates A (2005). Standardisation of spirometry. Eur Respir J.

