# Supplementary material for: Chronic exposure to diesel exhaust may cause small airway wall thickening without lumen narrowing: a quantitative computerized tomography study in Chinese diesel engine testers
Source: Part Fibre Toxicol. 2021 Mar 25;18:14. doi: 10.1186/s12989-021-00406-1 (PMC7992811; doi:10.1186/s12989-021-00406-1)
Supplement: Supplementary file 1 — Additional file 1: Supplemental Table 1. The anatomical diversity of airway measurements assessed in non-DETs and DETs separately with airway LB1 + 2 as the reference (n = 154)a. [file 12989_2021_406_MOESM1_ESM.docx]

Supplemental Table 1. The anatomical diversity of airway measurements assessed in non-DETs and DETs separately with airway LB1+2 as the reference (n=154)^a^

| Variable | Non-DET (n=76) | |  | DET (n=78) | |
| --- | --- | --- | --- | --- | --- |
|  | Median (Q1 - Q3) | *P* |  | Median (Q1 - Q3) | *P* |
| 6^th^ Wall area (mm^2^) |  |  |  |  |  |
| LB1+2 | 10.6 (7.5, 14.3) | REF |  | 12.7 (8.4, 16.3) | REF |
| LB9 | 9.5 (6.8, 14.6) | 0.228 |  | 12.9 (9.8, 17.5) | 0.248 |
| RB9 | 9.3 (5.8, 14.8) | 0.259 |  | 13.7 (10.0, 17.8) | 0.271 |
| RB1 | 8.3 (5.4, 12.7) | 0.013 |  | 10.1 (8.2, 13.0) | <0.001 |
| 6^th^ Lumen area (mm^2^) |  |  |  |  |  |
| LB1+2 | 8.7 (6.8, 11.0) | REF |  | 8.9 (7.0, 10.9) | REF |
| LB9 | 9.1 (6.7, 11.5) | 0.223 |  | 10.8 (8.4, 14.0) | <0.001 |
| RB9 | 8.4 (5.8, 10.4) | 0.536 |  | 10.2 (7.8, 12.9) | 0.008 |
| RB1 | 7.0 (4.9, 9.4) | <0.001 |  | 7.3 (6.0, 8.9) | <0.001 |
| 6^th^ Airway area (mm^2^) |  |  |  |  |  |
| LB1+2 | 19.9 (15.4, 23.3) | REF |  | 21.8 (15.5, 26.4) | REF |
| LB9 | 18.5 (13.6, 27.2) | 0.757 |  | 24.3 (19.1, 30.5) | 0.011 |
| RB9 | 17.9 (13.1, 24.7) | 0.274 |  | 23.8 (18.4, 29.7) | 0.042 |
| RB1 | 15.7 (12.0, 21.2) | <0.001 |  | 17.4 (14.5, 21.5) | <0.001 |
| 9^th^ Wall area (mm^2^) |  |  |  |  |  |
| LB1+2 | 2.3 (1.6, 3.1) | REF |  | 2.3 (1.8, 3.3) | REF |
| LB9 | 2.8 (1.9, 4.1) | 0.002 |  | 2.9 (2.2, 4.4) | <0.001 |
| RB9 | 2.6 (1.8, 4.3) | <0.001 |  | 2.6 (1.8, 4.1) | 0.240 |
| RB1 | 2.4 (1.7, 3.2) | 0.112 |  | 2.5 (1.9, 3.4) | 0.495 |
| 9^th^ Lumen area (mm^2^) |  |  |  |  |  |
| LB1+2 | 3.0 (2.2, 3.6) | REF |  | 3.0 (2.4, 3.7) | REF |
| LB9 | 3.5 (2.8, 4.9) | <0.001 |  | 3.9 (3.0, 5.0) | <0.001 |
| RB9 | 3.4 (2.7, 4.2) | 0.035 |  | 3.7 (2.7, 4.3) | 0.019 |
| RB1 | 2.8 (2.0, 3.6) | 0.152 |  | 2.9 (2.3, 3.5) | 0.151 |
| 9^th^ Airway area (mm^2^) |  |  |  |  |  |
| LB1+2 | 5.4 (4.4, 6.4) | REF |  | 5.5 (4.6, 7.1) | REF |
| LB9 | 6.7 (5.3, 7.8) | <0.001 |  | 7.1 (6.2, 8.9) | <0.001 |
| RB9 | 6.4 (5.2, 7.8) | <0.001 |  | 6.5 (5.5, 8.0) | 0.021 |
| RB1 | 5.2 (4.1, 6.8) | 0.980 |  | 5.6 (4.4, 6.8) | 0.717 |

Deﬁnition of abbreviations: DET = diesel engine tester; LB = left bronchus; RB = right bronchus; Q1 = lower quartile; Q3 = upper quartile; REF = reference.

^a^ Linear mixed effects model was used to assess the differences of natural log transformed measurements among different lung lobes with adjustment of age, BMI, smoking history, CT reconstruction method, and lung lobes. Areas were shown as median (Q1, Q3).
