# Supplementary material for: Chronic exposure to diesel exhaust may cause small airway wall thickening without lumen narrowing: a quantitative computerized tomography study in Chinese diesel engine testers
Source: Part Fibre Toxicol. 2021 Mar 25;18:14. doi: 10.1186/s12989-021-00406-1 (PMC7992811; doi:10.1186/s12989-021-00406-1)
Supplement: Supplementary file 3 — Additional file 3: Supplemental Table 3. The influencing factors for 6th generation airway dimensions in all study subjects (n = 154)a. [file 12989_2021_406_MOESM3_ESM.docx]

Supplemental Table 3. The influencing factors for 6^th^ generation airway dimensions in all study subjects (n=154)^a^

| Variable | 6^th^ WA (mm^2^) | | |  | 6^th^ LA (mm^2^) | | |  | 6^th^ AA (mm^2^) | | |  | 6^th^ WA% | | |
| --- | --- | --- | --- | --- | --- | --- | --- | --- | --- | --- | --- | --- | --- | --- | --- |
|  | Ratio | SE | *P* |  | Ratio | SE | *P* |  | Ratio | SE | *P* |  | Ratio | SE | *P* |
| Age (per 10 yr inc) | 1.07 | 1.04 | 0.079 |  | 1.16 | 1.04 | <0.001 |  | 1.11 | 1.03 | <0.001 |  | 0.97 | 1.02 | 0.061 |
| BMI (per 5 kg/m^2^) | 1.14 | 1.04 | <0.001 |  | 0.96 | 1.03 | 0.255 |  | 1.05 | 1.03 | 0.082 |  | 1.08 | 1.02 | <0.001 |
| Current smoker (vs never) | 1.10 | 1.08 | 0.229 |  | 1.07 | 1.07 | 0.321 |  | 1.08 | 1.06 | 0.195 |  | 1.02 | 1.04 | 0.682 |
| Former smoker (vs never) | 1.12 | 1.12 | 0.286 |  | 1.01 | 1.10 | 0.914 |  | 1.07 | 1.09 | 0.455 |  | 1.05 | 1.05 | 0.306 |
| Packyears (per 5 py) | 1.01 | 1.01 | 0.417 |  | 0.99 | 1.01 | 0.297 |  | 1.00 | 1.01 | 0.893 |  | 1.01 | 1.01 | 0.135 |
| Lung method (vs standard) | 1.10 | 1.11 | 0.323 |  | 0.94 | 1.09 | 0.500 |  | 1.02 | 1.08 | 0.838 |  | 1.09 | 1.05 | 0.078 |
| Location |  |  |  |  |  |  |  |  |  |  |  |  |  |  |  |
| LB1+2 | REF |  |  |  | REF |  |  |  | REF |  |  |  | REF |  |  |
| LB9 | 0.99 | 1.05 | 0.838 |  | 1.12 | 1.03 | <0.001 |  | 1.05 | 1.04 | 0.167 |  | 0.94 | 1.02 | 0.003 |
| RB9 | 0.99 | 1.05 | 0.858 |  | 1.05 | 1.03 | 0.172 |  | 1.02 | 1.04 | 0.657 |  | 0.98 | 1.02 | 0.215 |
| RB1 | 0.82 | 1.05 | <0.001 |  | 0.81 | 1.03 | <0.001 |  | 0.82 | 1.04 | <0.001 |  | 1.00 | 1.02 | 0.867 |
| DEE exposure (vs non-DET) | 1.25 | 1.06 | <0.001 |  | 1.11 | 1.06 | 0.065 |  | 1.18 | 1.05 | <0.001 |  | 1.07 | 1.03 | 0.031 |

Deﬁnition of abbreviations: BMI = body mass index; WA = wall area; LA = lumen area; AA = airway area; WA% = wall area percent; SE = standard error; LB = left bronchus; RB = right bronchus; REF = reference; DEE = diesel engine exhaust.

^a^ Linear mixed effects model assessed the associations of natural log transformed areas of wall, lumen, airway and wall area percent with eight factors. Ratio and SE were exponentials of β and standard error for each factor in linear mixed effects model.
