# Supplementary material for: Chronic exposure to diesel exhaust may cause small airway wall thickening without lumen narrowing: a quantitative computerized tomography study in Chinese diesel engine testers
Source: Part Fibre Toxicol. 2021 Mar 25;18:14. doi: 10.1186/s12989-021-00406-1 (PMC7992811; doi:10.1186/s12989-021-00406-1)
Supplement: Supplementary file 4 — Additional file 4: Supplemental Table 4. The influencing factors for 9th generation airway dimensions in all study subjects (n = 154)a. [file 12989_2021_406_MOESM4_ESM.docx]

Supplemental Table 4. The influencing factors for 9^th^ generation airway dimensions in all study subjects (n=154)^a^

| Variable | 9^th^ WA (mm^2^) | | |  | 9^th^ LA (mm^2^) | | |  | 9^th^ AA (mm^2^) | | |  | 9^th^ WA% | | |
| --- | --- | --- | --- | --- | --- | --- | --- | --- | --- | --- | --- | --- | --- | --- | --- |
|  | Ratio | SE | *P* |  | Ratio | SE | *P* |  | Ratio | SE | *P* |  | Ratio | SE | *P* |
| Age (per 10 yr inc) | 0.96 | 1.04 | 0.296 |  | 1.08 | 1.03 | 0.007 |  | 1.02 | 1.02 | 0.439 |  | 0.94 | 1.03 | 0.042 |
| BMI (per 5 kg/m^2^) | 1.13 | 1.04 | 0.002 |  | 1.04 | 1.03 | 0.117 |  | 1.09 | 1.02 | <0.001 |  | 1.04 | 1.03 | 0.220 |
| Current smoker (vs never) | 0.94 | 1.09 | 0.422 |  | 0.91 | 1.06 | 0.093 |  | 0.90 | 1.04 | 0.020 |  | 1.04 | 1.06 | 0.555 |
| Former smoker (vs never) | 1.01 | 1.12 | 0.942 |  | 0.86 | 1.08 | 0.060 |  | 0.91 | 1.06 | 0.133 |  | 1.11 | 1.09 | 0.226 |
| Packyears (per 5 py) | 1.00 | 1.01 | 0.908 |  | 1.00 | 1.01 | 0.663 |  | 1.00 | 1.01 | 0.739 |  | 1.00 | 1.01 | 0.925 |
| Lung method (vs standard) | 1.08 | 1.11 | 0.470 |  | 1.10 | 1.08 | 0.194 |  | 1.10 | 1.06 | 0.096 |  | 0.98 | 1.08 | 0.818 |
| Location |  |  |  |  |  |  |  |  |  |  |  |  |  |  |  |
| LB1+2 | REF |  |  |  | REF |  |  |  | REF |  |  |  | REF |  |  |
| LB9 | 1.25 | 1.05 | <0.001 |  | 1.28 | 1.04 | <0.001 |  | 1.28 | 1.04 | <0.001 |  | 0.98 | 1.02 | 0.453 |
| RB9 | 1.17 | 1.05 | <0.001 |  | 1.13 | 1.04 | 0.002 |  | 1.17 | 1.04 | <0.001 |  | 1.01 | 1.02 | 0.756 |
| RB1 | 1.09 | 1.05 | 0.106 |  | 0.92 | 1.04 | 0.042 |  | 0.99 | 1.04 | 0.77 |  | 1.09 | 1.02 | <0.001 |
| DEE exposure (vs non-DET) | 1.12 | 1.07 | 0.090 |  | 1.09 | 1.05 | 0.067 |  | 1.10 | 1.04 | 0.005 |  | 1.02 | 1.05 | 0.744 |

Deﬁnition of abbreviations: BMI = body mass index; WA = wall area; LA = lumen area; AA = airway area; WA% = wall area percent; SE = standard error; LB = left bronchus; RB = right bronchus; REF = reference; DEE = diesel engine exhaust.

^a^ Linear mixed effects model assessed the associations of natural log transformed areas of wall, lumen, airway and wall area percent with eight factors. Ratio and SE were exponentials of β and standard error for each factor in linear mixed effects model.
