# Supplementary material for: Chronic exposure to diesel exhaust may cause small airway wall thickening without lumen narrowing: a quantitative computerized tomography study in Chinese diesel engine testers
Source: Part Fibre Toxicol. 2021 Mar 25;18:14. doi: 10.1186/s12989-021-00406-1 (PMC7992811; doi:10.1186/s12989-021-00406-1)
Supplement: Supplementary file 5 — Additional file 5: Supplemental Table 5. The effect of diesel exhaust exposure status on 6th generation airway dimensions in all study subjects (n = 154)a. [file 12989_2021_406_MOESM5_ESM.docx]

Supplemental Table 5. The effect of diesel exhaust exposure status on 6^th^ generation airway dimensions in all study subjects (n=154)^a^

| Variable | Non-DET (n=76) | DET (n=78) | Ratio (95CI%) | *P* |
| --- | --- | --- | --- | --- |
| Wall area (mm^2^) |  |  |  |  |
| LB1+2 | 10.6 (7.5, 14.3) | 12.7 (8.4, 16.3) |  |  |
| LB9 | 9.5 (6.8, 14.6) | 12.9 (9.8, 17.5) |  |  |
| RB9 | 9.3 (5.8, 14.8) | 13.7 (10.0, 17.8) |  |  |
| RB1 | 8.3 (5.4, 12.7) | 10.1 (8.2, 13.0) |  |  |
| All | 9.2 (8.4, 10.1) | 11.5 (10.5, 12.7) | 1.25 (1.11, 1.41) | <0.001 |
| Lumen area (mm^2^) |  |  |  |  |
| LB1+2 | 8.7 (6.8, 11.0) | 8.9 (7.0, 10.9) |  |  |
| LB9 | 9.1 (6.7, 11.5) | 10.8 (8.4, 14.0) |  |  |
| RB9 | 8.4 (5.8, 10.4) | 10.2 (7.8, 12.9) |  |  |
| RB1 | 7.0 (4.9, 9.4) | 7.3 (6.0, 8.9) |  |  |
| All | 7.9 (7.3, 8.5) | 8.7 (8.0, 9.5) | 1.11 (0.99, 1.23) | 0.065 |
| Airway area (mm^2^) |  |  |  |  |
| LB1+2 | 19.9 (15.4, 23.3) | 21.8 (15.5, 26.4) |  |  |
| LB9 | 18.5 (13.6, 27.2) | 24.3 (19.1, 30.5) |  |  |
| RB9 | 17.9 (13.1, 24.7) | 23.8 (18.4, 29.7) |  |  |
| RB1 | 15.7 (12.0, 21.2) | 17.4 (14.5, 21.5) |  |  |
| All | 17.7 (16.5, 19.0) | 20.8 (19.3, 22.4) | 1.18 (1.07, 1.29) | <0.001 |

Deﬁnition of abbreviations: DET = diesel engine tester; CI = confidence interval; LB = left bronchus; RB = right bronchus.

^a^ Linear mixed effects model assessed differences of natural log transformed areas of wall, lumen, and airway between non-DETs and DETs with adjustment of age, BMI, smoking history, CT reconstruction method, and lung lobes. Indices were shown as median (Q1, Q3). Ratio = e^β^, 95%CI = e^(β ± 1.96×Se)^ . Descriptive statistics for all were exponentials of least square means and 95%CIs that were calculated based on natural log transformed data using linear mixed effects model with adjustment for covariates listed above and may be regarded as an overall level of airway dimension indices based on the four sampled airways of 6^th^ generation.
