# Supplementary material for: Chronic exposure to diesel exhaust may cause small airway wall thickening without lumen narrowing: a quantitative computerized tomography study in Chinese diesel engine testers
Source: Part Fibre Toxicol. 2021 Mar 25;18:14. doi: 10.1186/s12989-021-00406-1 (PMC7992811; doi:10.1186/s12989-021-00406-1)
Supplement: Supplementary file 7 — Additional file 7: Supplemental Table 7. Mediation effect of airway wall thickening (measured by wall area percent) on associations between diesel exhaust exposure and FEV1 in all subjects (n = 154)a. [file 12989_2021_406_MOESM7_ESM.docx]

Supplemental Table 7. Mediation effect of airway wall thickening (measured by wall area percent) on associations between diesel exhaust exposure and FEV1 in all subjects (n=154)^a^

| Variable | Model 1 | | |  | Model 2 | | |
| --- | --- | --- | --- | --- | --- | --- | --- |
|  | β | SE | *P* |  | β | SE | *P* |
| DE exposure (vs non-DET) | -0.153 (C) | 0.065 | 0.020 |  | -0.124 (C') | 0.065 | 0.059 |
| 6^th^ wall area percent (1% ) |  |  |  |  | -0.0093 | 0.0038 | 0.015 |
| R^2^ | 0.46 |  |  |  | 0.48 |  |  |
| PM^b^ |  |  |  |  | 0.20 |  |  |
| P_perm_^b^ |  |  |  |  | 0.028 |  |  |

Deﬁnition of abbreviations: DEE = diesel engine exhaust; SE = standard error; BMI = body mass index; R^2^ = determination coefficient; PM = proportion mediated; Pperm = permuted P value.

^a^ GLM was used to assess association between DEE exposure and FEV1 with adjustment of age, height, BMI, and smoking history. R^2^ was calculated to describe the proportion of variation of FEV1 that can be explained by independent variables.

^b^ In mediation analysis, the C coefficient denotes the direct effect of DE exposure on FEV1, without controlling for airway wall thickening (e.g., wall area percent, mediator). The C' coefficient denotes the direct effect of DE exposure on FEV1, controlling for airway wall thickening (mediator). The proportion mediated is equal to delta C (i.e., C-C') divided by C. We took a permutation-based method to assess whether the proportion mediated was statistically significant or not. The relationship between FEV1 and the vector of independent variables was permuted for 500 times. Each permutated database allowed the association analysis of FEV1 with DE exposure and other covariates without and with including wall area percent to calculate the C and C'. Permutation was conducted for 1000 times to generate the distribution of C-C' under null hypothesis of no mediation. Value of C-C' calculated using observed data (-0.03) was compared to the distribution generated by permutation and P_perm_ was calculated as the number of permuted databases generating a C-C' that is smaller than observed value (n=14) divided by 500. Thus, P_perm_ was 0.028 in this case.
